# Cryochemical Production of Drug Nanoforms: Particle Size and Crystal Phase Control of the Antibacterial Medication 2,3-Quinoxalinedimethanol-1,4-dioxide (Dioxidine)

**DOI:** 10.3390/nano11061588

**Published:** 2021-06-17

**Authors:** Tatyana I. Shabatina, Yurii N. Morosov, Andrey V. Soloviev, Andrey V. Shabatin, Olga I. Vernaya, Michail Y. Melnikov

**Affiliations:** 1 Department of Chemistry, M.V. Lomonosov Moscow State University, Leninskie Gori Build 1/3, 119991 Moscow, Russia; yunmor@mail.ru (Y.N.M.); andr_solovyov@mail.ru (A.V.S.); olga_vernaya@mail.ru (O.I.V.); melnikov46@mail.ru (M.Y.M.); 2 Department of Chemistry, Faculty of Fundamental Sciences, Bauman Moscow State Technical University, 2nd Bauman Str. 5, 105905 Moscow, Russia; 3 A.N. Frumkin Institute of Physical Chemistry and Electrochemistry RAS, Lenin Prospect, 31 Build 4, 119071 Moscow, Russia; 5dr.on5@mail.ru

**Keywords:** drug nanoforms, cryochemical synthesis of nanocrystals, particle size reduction, crystal structure modification, antibacterial drug dioxidine

## Abstract

Increasing the effectiveness of known, well-tested drugs is a promising low-cost alternative to the search for new drug molecular forms. Powerful approaches to solve this problem are (a) an active drug particle size reduction down to the nanoscale and (b) thermodynamically metastable but kinetically stable crystal modifications of drug acquisition. The combined cryochemical method has been used for size and structural modifications of the antibacterial drug 2,3-quinoxalinedimethanol-1,4-dioxide (dioxidine). The main stage of the proposed technique includes the formation of a molecular vapor of the drug substance, combined with a carrier gas (CO_2_) flow, followed by a fast condensation of the drug substance and CO_2_ molecules on a cooled-by-liquid nitrogen surface of preparative cryostate. It was established that the molecular chemical structure of the drug substance remained unchanged during cryochemical modification; however, it led to a significant decrease of the drug particles’ size down to nanosizes and changes in the crystal structures of the solid drug nanoforms obtained. Varying carrier gas (CO_2_) flow led to changes in their solid phase composition. A higher dissolution rate and changes in antibacterial activity were demonstrated for cryomodified dioxidine samples in comparison to the properties of the initial pharmacopeia dioxidine.

## 1. Introduction

The need to develop new drug substances and medications is determined by the fact that, today, many well-known, long-established drugs do not meet modern medical requirements. However, the creation and testing of new molecular forms of drugs (drug discovery) require not only huge material costs, reaching several billions of dollars, but also many years spent on conducting various clinical and preclinical tests. From this point of view, another approach is more promising and consists of an increase in the effectiveness of known medicines and improved methods of their targeted delivery (drug delivery). This can be achieved, for example, by reducing drug particle size down to the nanoscale state [1], as well as by obtaining new or previously known thermodynamically metastable, but kinetically stable, polymorphic crystal modifications [2]. The size is also of primary importance because the size parameters largely determine the bioavailability of the drug [3].

The different physical and chemical methods of drug nanoform production can be divided into two main groups: “top-down” and “bottom-up”; both have been used in pharmaceutical practice [4,5,6]. The “top-down” methods include the grinding of the larger drug substance particles via mechanical action. This method uses different techniques of dry and wet crushing of the initial materials in special kinds of mills [7], including the cryogenic milling processes [8]. Homogenization of dispersions under ordinary and high-pressure conditions and different size selecting techniques using nanoporous membranes, filters, etc., also belong to this group [9,10,11].

On the other hand, the “bottom-up” approach consists of a conversion of the initial pharmacopoeia substance in the molecular homogeneous state and then the creation of the conditions for homogeneous or heterogeneous nucleation and new nanophase particle growth [12,13,14]. Examples of such techniques include methods that replace the solvents [15], the use of supercritical fluids [16,17,18], freeze-drying and spray drying techniques [19,20,21], and cryochemical synthesis [22,23,24].

Particle size is one of the key aspects determining pharmaceutical preparation bioavailability [25]. For example, decreasing the size of an antigonadotropic drug diazole in water suspension from the size of 10 microns, for the particles of the initial preparation, to a particle size of 169 nanometers, for the modified drug substance, led to increasing the bioavailability value from (5.1 ± 1.9)% up to (82.3 ± 10.1)% [26]. Bioavailability enhancement may allow investigators to decrease the therapeutic dose for the drug preparation and could reduce possible side effects. Decreasing particles size could lead to changes in drug solubility and raise the possibility of small drug particle penetration through biological barriers of the organisms [5,27].

The effect of polymorphism—the existence of a substance of the same chemical structure in several crystalline forms—is currently the subject of close attention by both scientists and health surveillance services because, when the polymorphic modification changes, both the physicochemical (melting point, density, thermodynamic stability, solubility, and dissolution rate) and the therapeutic (bioequivalence, bioavailability, etc.) characteristics of drugs also change [28,29,30,31,32]. Thus, the ability to control the phase composition of crystalline drugs is extremely important for biomedical applications.

The aim of this work was to develop a cryochemical production method for drug nanoforms with a size of several nanometers and new structural characteristics using the antibacterial drug 2,3-quinoxalinedimethanol-1,4-dioxide (dioxidine) as an example.

## 2. Materials and Characterization Methods

### 2.1. Materials

**Scheme 1:**
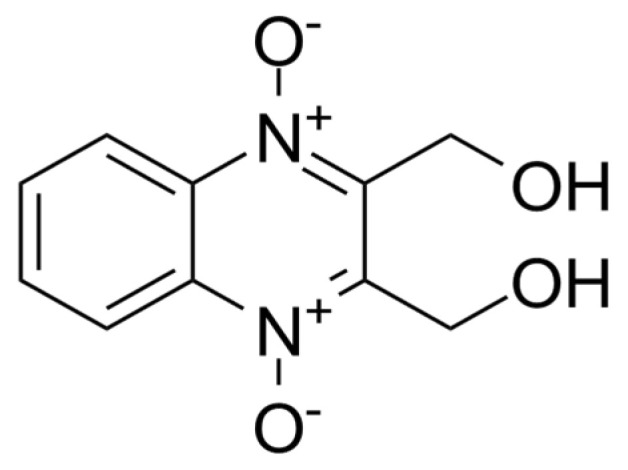
Chemical Structure of dioxidine.

**Figure 1:**
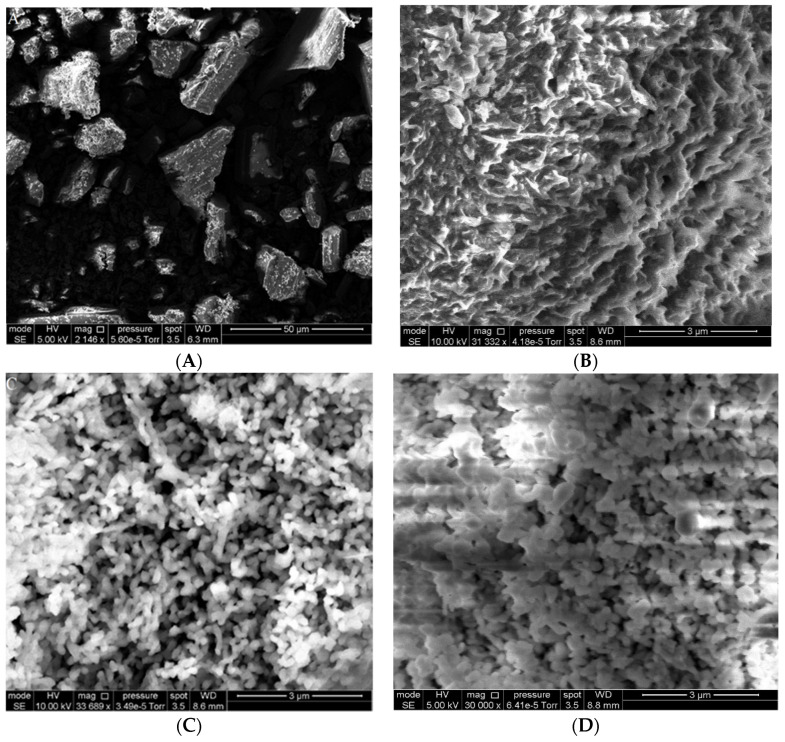
SEM images of the pharmacopoeia dioxidine (**A**) and of the cryomodified dioxidine samples obtained in the absence of a gas carrier (CO_2_) flow (**B**), with a gas carrier (CO_2_) flow of 7.0 × 10^16^ molecules s^−1^ cm^−2^ (**C**), with a carrier gas (CO_2_) flow of 7.0 × 10^17^ molecules s^−1^ cm^−2^ (**D**).

2,3-Quinoxalinedimethanol-1,4-dioxide (dioxidine), produced by Mir-Pharma (Moscow, Russian Federation) (98.9%), was used without further purification. The pharmaceutical dioxidine exists in hydrate form, whose crystal structure is known (SOKGAA Cambridge Structural Database file) [33,34]. The average particle size, according to SEM image (Figure 1A) and BET-surface area measurements, is 10–50 micrometers. The chemical structure of the dioxidine molecule is in Scheme 1.

### 2.2. Sample Preparation: Cryochemical Synthesis of Dioxidine Nanoforms

Cryochemical modification of dioxidine was performed by a combined method using a specially designed preparative cryostate. The process included sublimation of the initial dioxidine, inserting its molecular beam in the flow of an inert gas (carrier carbon dioxide (CO_2_), low), and temperature condensation of the beams of the dioxidine drug substance and the carrier-gas CO_2_ on a cooled-by-liquid nitrogen pyrex glass surface. The cryostat was equipped with a heated stainless steel grid (the size of one hole was 40 micrometers, the steel wire was 30 micrometers), which was used for the sublimation of the drug dioxidine. The design of cryostate is described in details in the patent [35]. As a carrier gas, carbon dioxide (CO_2_) was used, which is almost completely condensed on the cryostate surface, which is cooled by liquid nitrogen. The scheme of the original setup is shown in the Supplement Materials Section, Figure S1.

During cryochemical modification of dioxidine, the variable parameter was the value flow rate of the carrier gas—CO_2_. Other experimental conditions, namely, the temperature of the metal grid (140 °C), the temperature of the cooled surface (−196 °C), the geometric location of the sublimation unit, and the nozzles for supplying carbon dioxide in the cryostat, were unchanged during all experiments.

### 2.3. Characterization Techniques

#### 2.3.1. UV-Vis

A UV-Visible-NIR double beam spectrophotometer “Jasco V-770” (JASCO Corporation, Tokyo, Japan) was used to record the spectra of dioxidine in aqueous solutions and spectrophotometric determination of dioxidine concentrations in solutions in the range of 200–900 nm at room temperature using optical quartz cuvettes (l = 1–2 mm). In most cases, the solutions were saturated by the inert gas argon.

#### 2.3.2. FTIR-Spectroscopy

FTIR spectra of the solid cryomodified samples were recorded using an FTIR spectrometer Tensor II (Bruker GmbH, Mannheim, Germany) with an attenuated total reflection (ATR) module A225/Q platinum.

#### 2.3.3. X-ray Powder Diffraction

X-ray powder diffraction patterns were recorded with Empyrean (Panalytical, Almelo, The Netherlands) in Bragg−Brentano mode using non-monochromated Cu Kα radiation in a 4–40° 2Θ region. The averaged size of microcrystallites was estimated from the diffraction peak broadening using Scherrer formula, $d=\frac{K*\lambda }{\beta *cos\theta }$, where *d*—the average size of particles, *λ*—x-ray wavelength, *β*—peak width at half maximum (FWHM), *θ*—diffraction angle (Bragg angle), and *K*—dimensionless particle form factor (Scherrer factor). For spherical particles *K* = 0.94 was used [36].

#### 2.3.4. Scanning Electron Microscopy

SEM images of the initial and cryomodified dioxidine particles were recorded using an electron microscope, FEI QUANTA 650 FEG (Thermo Fisher Scientific, Hillsboro, OR, USA), Collective Facilities Center of A.N. Frumkin Institute of Physical Chemistry and Electrochemistry RAS.

#### 2.3.5. BET-Specific Surface Area Measurements

The size of the initial and modified dioxidine particles was determined using the values of the specific surface area of the obtained samples, measured by low temperature argon adsorption. The BET measurements of the values of the specific surface area (S, m^2^/g) of samples were defined using a surface analyzer assembled with a gas-chromatograph “Chrom 5” [37].

Average dioxidine particle diameters (d) of the samples were calculated as d = 6/(ρS), where S (m^2^/g) is a specific area of the obtained samples and ρ is a specific density (g/m^3^) of dioxidine substance. The specific surface area of the cryomodified dioxidine samples was varied from 50 up to 100 m^2^/g.

#### 2.3.6. Antibacterial Tests

The antibacterial activities of different dioxidine forms were determined via a disk-diffusion test using compressed tablets (100 mg, d = 6 mm, 4900 kPa) of the initial pharmacopoeia dioxidine and cryomodified sample (obtained at a carrier gas flow of 6.97 × 10^17^ molecules s^−1^ cm^−2^) [38]. Bacteria cells from the collection of the Department of Biology, Moscow State University (*E. coli 52*, *S. aureus 144*, *M. cyaneum 98*, and *B. cereus 9*) were served as test cultures. The experiments were performed in Petri dishes containing 20 mL of nutrient agar medium dried for one day (average layer thickness, 4 mm). The bacterial cells of the test cultures in an amount of 10^8^ were seeded onto each 90-mm agar dish for further disc-diffusion experiments. The inhibition zones of the test culture were measured 24 h after the start of incubation. Statistically reliable results were obtained by nine-fold repetition measurements of the inhibition zones (ZOI) of bacterial strains growth for each series of samples.

#### 2.3.7. Statistical Analysis

All data are reported as mean with standard deviation (±SD). For statistical analysis, the Excel *T*-*TEST* function was used, and statistically significant differences were described as *p*-values less than 0.05 (*p* < 0.05).

## 3. Results and Discussion

SEM microphotographs of samples of the initial pharmacopeia dioxidine medication (Figure 1A) and cryochemically obtained dioxidine nanoforms (Figure 1B–D) are presented in Figure 1. The particle size of cryomodified samples using the combined cryochemical method with carrier gas (CO_2_) flow rates of 10^16^–7 × 10^17^ molecules s^−^^1^ cm^−^^2^ led to a significant reduction in size of the drug substance particles by more than 100–1000 times from 10–50 micrometers down to 50–100 nm. Such cryomodification of drug substance could lead to a change of the different physicochemical properties of the samples obtained and could improve the general bioavailability of the dioxidine medication.

**Figure 2:**
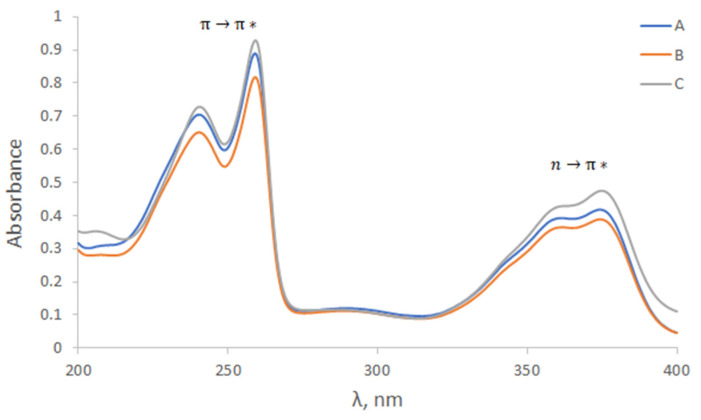
UV-Vis spectra of aqueous solutions of dioxidine: A—pharmacopoeia dioxidine (0.0029 mg/mL), B—cryochemically modified dioxidine obtained at a zero carrier gas flow (0.0028 mg/mL), C—cryochemically modified dioxidine obtained at a carrier gas flow of 6.97 × 10^17^ molec s^−1^ cm^2^ (0.0031 mg/mL).

UV/Vis spectra of aqueous solutions of the initial and cryochemically modified dioxidine are presented in Figure 2. The spectra contain an intense doublet band with maxima at 241 and 259 nm due to the π→π * electronic transition of the aromatic system, and a low-intensity band with a maximum at 375 nm due to the n→π * electronic transition of the unshielded paired electrons of nitrogen atoms of the dioxidine molecule, which is in accordance with literature data [39]. According to UV/Vis data in the course of cryochemical modification of dioxidine, there were no changes in its molecular structure.

**Figure 3:**
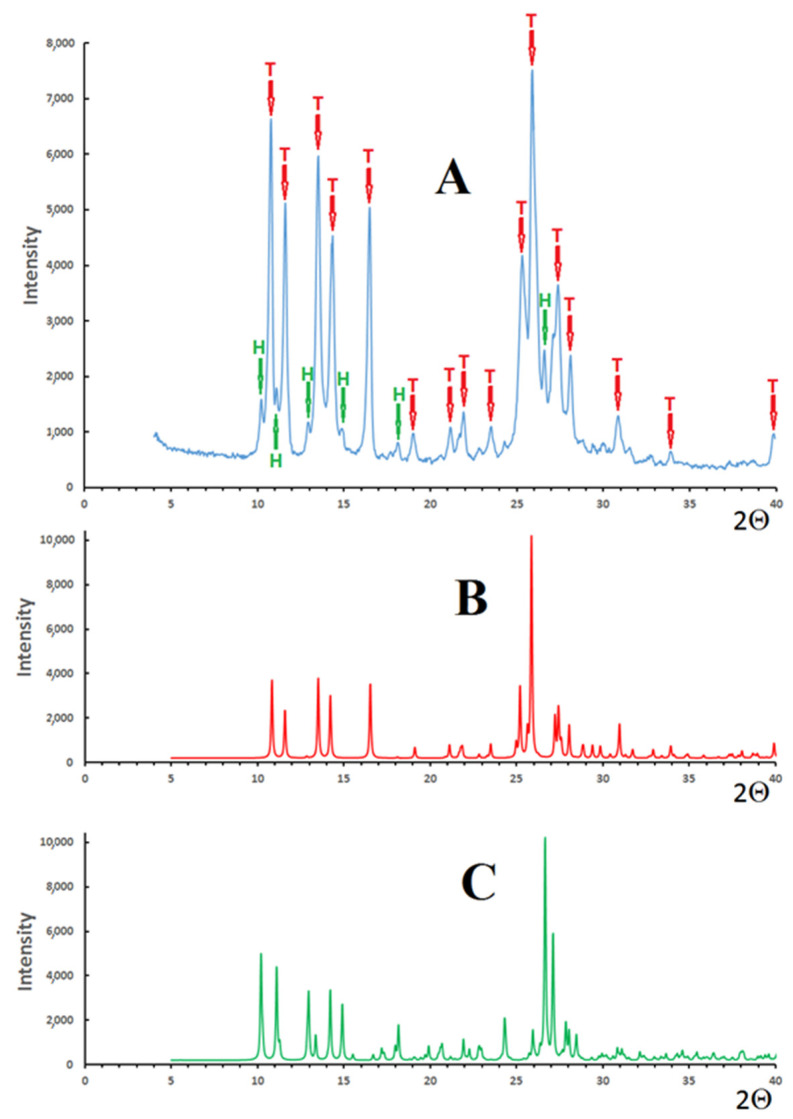
(**A**) experimentally recorded diffractogram of dioxidine obtained at zero flow of carrier gas (CO_2_), (**B**) theoretical diffractogram of dioxidine triclinic phase (T-phase) [41], (**C**) theoretical diffractogram of 1/3 dioxidine crystalline hydrate (H-phase) [41].

**Figure 4:**
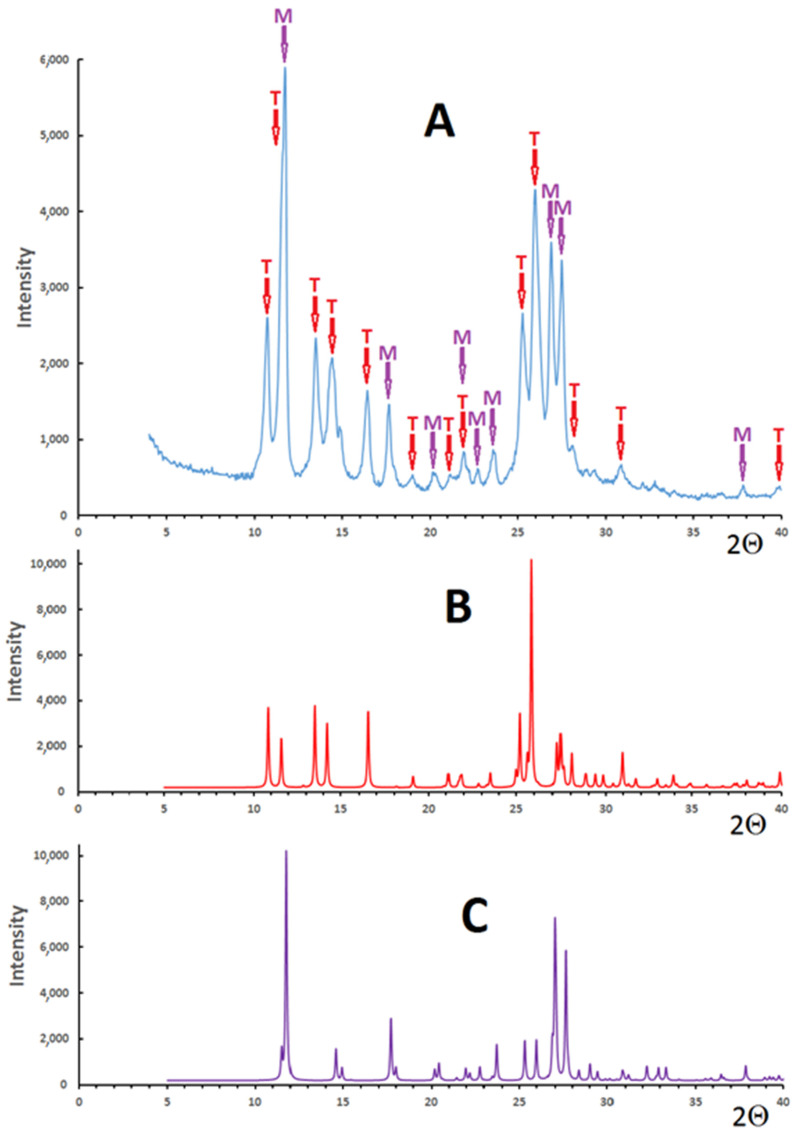
(**A**) experimentally recorded diffractogram of dioxidine obtained with a carrier gas (CO_2_) flow of 7.0 × 10^16^, (**B**) theoretical diffractogram of dioxidine triclinic phase (T-phase) [41], (**C**) theoretical diffractogram of dioxidine monoclinic phase (M-phase) [41].

**Figure 5:**
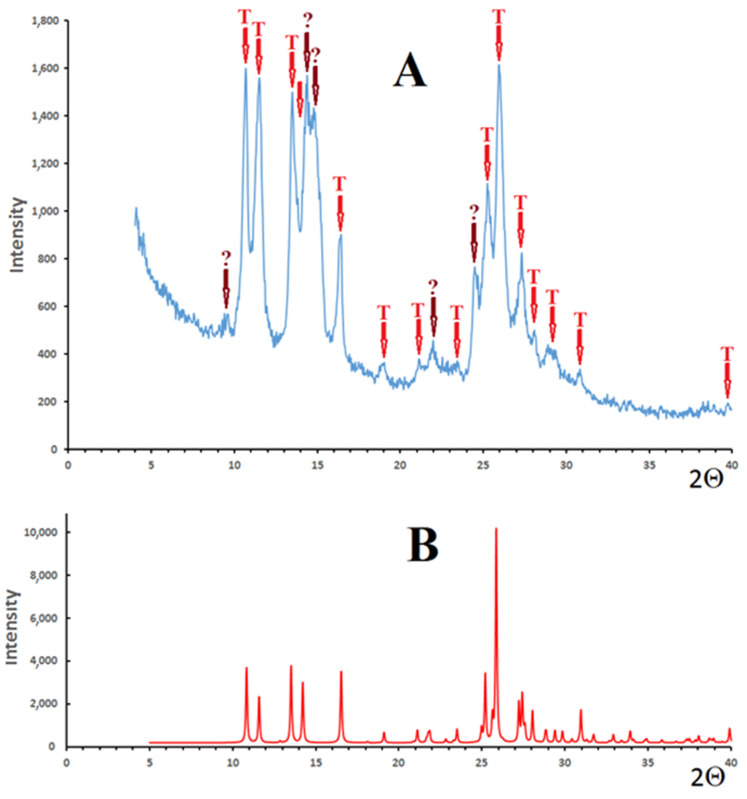
(**A**) experimentally recorded diffractogram of dioxidine obtained with a carrier gas flow (CO_2_) 7.0 × 10^17^ (?—unknown phase N of dioxidine), (**B**) theoretical diffractogram of the triclinic phase of dioxidine (T-phase) [41].

At the same time, cryochemically modified samples possess crystal structures that were significantly different from the crystal structure of the initial pharmaceutical medication dioxidine. The examples of the experimentally recorded X-ray diffraction of cryochemically converted dioxidine patterns, obtained using different carrier gas (CO_2_) flow rates are presented in Figure 3, Figure 4 and Figure 5. The crystallographic parameters of previously obtained anhydrous polymorphic modifications of dioxidine (Triclinic (T-phase) and monoclinic (M-phase) [40]) and its crystal hydrates (monohydrate (1:1) [41] and hydrate (1:3) (H-phase) [40]) and the molecular packing (3D-fragments) in these crystal structures, as well as the calculated powder diffraction patterns for these phases, are shown in Supplementary Materials, Figures S2–S9. Data on the phase composition and on the average particle sizes of cryomodified dioxidine nanoforms obtained by the proposed cryochemical method are presented in Table 1. These data indicate a dependence of the size and structural characteristics of the obtained nanoforms on the value of the carrier gas flow (CO_2_).

The sample obtained by the sublimation-condensation of dioxidine in the absence of a carrier gas flow contains an almost pure Triclinic (T) phase with small additions of the H-phase (1/3 crystalline hydrate of dioxidine) [41]. Previously, to obtain the T-phase, it was required to heat the dioxidine crystal hydrate obtained by freeze-drying at 120 °C over 8 h [41]. During such temperature exposure, the sample loses the properties of nanoforms because the particles of the drug are enlarged.

Samples obtained at small and medium carrier gas flows contain only Triclinic (T) and Monoclinic (M) phases, and the ratio of these phases in the experiment changes when the carrier gas flow varies—with an increase in the flow, the M-phase content increases from 33% at a flow of 1.4 × 10^16^ molecules s^−1^ cm^−2^ to 60% at a flow of 7.0 × 10^16^ molecules s^−1^ cm^−2^.

Samples obtained at high values of the carrier gas flow (7 × 10^17^ molecules s^−1^ cm^−2^), along with the T-phase, contain a new unidentified phase (N), and the phase composition of these samples ceases to depend on the value of the carrier gas flow.

The average particle size of cryochemically modified dioxidine samples, measured by chromatographic determination of the amount of adsorbed inert gas argon and specific surface area (S, m^2^/g) of the samples (see Section 2.3.5), decreases with an increase of carbon dioxide flow rate—from (414 ± 80) nm at a minimal, close to zero CO_2_ gas flow rate, down to (108 ± 22) nm at a carrier gas (CO_2_) flow rate of 7 × 10^16^ molecules s^−1^ cm^−2^ and to (51 ± 10) nm at a maximal carrier gas flow rate of 7 × 10^17^ molecules s^−1^ cm^−2^ (see Table 1).

The dependence of the average particle size of cryochemically modified dioxidine on the value of the carrier gas flow can be explained by the competition of nucleation mechanisms: heterogeneous surface and homogeneous gas-phase condensation. In the absence of a carrier gas, the stationary concentration of dioxidine molecules (1.5 × 10^21^ molecules/m^3^) corresponds to the conditions of the “Maxwell gas”, whose thermal conductivity does not depend on the concentration. Therefore, the heat exchange between the cooled surface and the molecular flow of dioxidine is carried out by the mechanism of thermal conductivity. Possibly, the cooling rate of the dioxidine molecular flow is insufficient for effective homogeneous gas-phase nucleation, so the nucleation and growth of dioxidine crystallites were carried out by a less effective surface heterogeneous mechanism.

The use of a carrier gas, CO_2_, leads to a significantly faster cooling of the dioxidine molecular flow as it moves towards a cold surface. It is achieved both by convective mixing of molecular flows—the “ hot “ flow of dioxidine and the relatively cold flow of carbon dioxide—and by the higher thermal conductivity of the molecular mixture of dioxidine and CO_2_.

An increase in the value of the carrier gas flow leads to more efficient cooling of the dioxidine molecular flow. In this case, the contribution of the homogeneous gas-phase mechanism to the processes of nucleation and crystal growth increases, which leads to a decrease in the average particle size of cryochemically modified dioxidine. At a certain value of the carrier gas flow, the contribution of the homogeneous gas-phase mechanism approach is about one hundred percent, and its further increase does not lead to a change in the average particle size, which is consistent with experimental data. These statements were confirmed experimentally by the SEM microphotographs of samples of cryochemically obtained dioxidine nanoforms shown in Figure 1.

A microphotograph of a cryochemically modified dioxidine sample obtained in the absence of a carrier gas (Figure 1A) does not show individual particles, but the surface of the sample has a complex relief with many small details. This pattern indicates that the nucleation and growth of a new phase in the absence of a carrier gas (CO_2_) flow proceeds through a heterogeneous surface mechanism. The characteristic size of the terrain irregularities is 200–400 nm, which is consistent with the estimate of the average particle size of the sample determined by the adsorption method (414 ± 80 nm).

The SEM micrograph of a sample of cryochemically modified dioxidine obtained with a carrier gas flow of 7.0 × 10^16^ molecules s^−1^ cm^2^ (Figure 1C) clearly shows individual particles whose size lies in the range of 100–200 nm, with an average particle size d = (120 ± 22) nm, while the average particle size determined by the adsorption method for this sample is (108 ± 22) nm. Taking into consideration that the resolution of the microscope does not allow recognition of the subtle details of the observed morphology of nanoparticles, it can be argued that, for this sample, the SEM results correlate well with the estimate of the average particle size obtained by adsorption method.

The SEM micrograph of a cryochemically modified dioxidine sample obtained with a carrier gas flow of 6.97 × 10^17^ molecules s^−1^ cm^−2^ (Figure 1D) shows well separated individual particles whose size lies within 50–100 nm, with an average particle size d = (69 ± 10) nm, while the average particle size determined by the adsorption method for this sample is (51 ± 10) nm. Probably, the nanoparticles formed under these conditions, which have a high surface energy, partially agglomerate, preserving their individuality to some extent.

The size of cryochemically modified dioxidine nanocrystallites, estimated on the basis of the broadening of X-ray peaks using the Scherer formula [37] (see Section 2.3.3), were equal to (88 ± 16) nm for the T-phase and (76 ± 15) nm for the H-phase crystallites obtained at minimal CO_2_ gas-flow rate, (33 ± 5) nm for T-phase and (60 ± 8) nm for M-phase crystallites obtained at CO_2_ gas-flow rate of 7 × 10^16^ molecules s^−1^ cm^−2^, and (40 ± 6) nm for T-phase crystallites obtained at CO_2_ gas-flow rate of 7 × 10^17^ molecules s^−1^ cm^−2^, confirming the tendency of reducing cryomodified dioxidine particle size by using sufficient gas CO_2_ flow. It should be mentioned that the data obtained using X-ray peaks broadening give us the sizes of the coherent scattering regions (CSR) of the dioxidine crystallites obtained. Dioxidine particles can be complex and consist of multiple CSRs; several particles can form conglomerates and splices. This leads to the fact that the particle sizes determined by the scanning electron microscopy (SEM) method are larger in many cases than the CSR dimensions obtained using X-ray peaks broadening.

An increase in the value of the carrier gas flow leads to a more efficient cooling of the molecular flow of dioxidine, primarily due to convective mixing. At the same time, the contribution of a homogeneous gas-phase mechanism to the processes of nucleation and crystal growth increases, and this leads to a decrease in the average particle size of cryochemically modified dioxidine. At a certain value of the carrier gas flow, the contribution of the homogeneous gas-phase mechanism approaches one hundred percent, and its further increase does not lead to a change in the average particle size, which is consistent with experimental data.

The effect of the carrier gas (CO_2_) flow on the crystal phase state of cryochemically modified dioxidine can be explained by several considerations. Anhydrous modifications of dioxidine—monoclinic or M-phase and triclinic or T-phase—are characterized by different organization of the system of intermolecular hydrogen bonds [41]. The triclinic or T-phase of dioxidine is characterized by the presence of infinite chains of molecules connected by intermolecular hydrogen bonds, with two N-oxide and two hydroxyl groups forming cross-linked hydrogen bonds (N→O....H-O) with two neighboring dioxidine molecules.

In the monoclinic or M-phase, the system of hydrogen bonds is arranged in a more complex way. One of the N-oxide groups of the dioxidine molecule does not participate in the formation of hydrogen bonds, and the connection of the dioxidine molecule with two neighboring ones is carried out by forming a three-center hydrogen bond between the hydroxyl group of this molecule and the hydroxyl group of one neighboring molecule and the N-oxide group of another neighboring molecule (N→O....H-O....H-O) [41]. The second hydroxyl group of this molecule forms a two-center hydrogen bond with the hydroxyl group of the neighboring molecule, which participates in the formation of a three-center hydrogen bond with the next molecule [41].

The primary process of new phase nucleation is the formation of dimers of molecules, which are then enlarged by attaching additional molecules and could form viable nuclei of a new phase. From this point of view, “natural” dimers can be distinguished in the crystal structure of the T-phase, in which dioxidine molecules are bound by cross-linked hydrogen bonds (N→O....H-O). It is likely that these “natural” dimers are the predominant dimer form in the gas phase. Additional dioxidine molecules are added to the “natural” dimer by saturating the existing free active centers (N→O, H-O). Thus, the molecular assembly in the T-phase crystal structure does not require any structural rearrangements.

In the M-phase, the structural unit includes four individual dioxidine molecules, which can be considered, not as a dimer, but as a tetramer superstructure. Thus, the processes of M-phase nucleation require certain structural transformations involving “natural” dimers. Therefore, it is likely that the T-phase nucleation is facilitated by a heterogeneous surface mechanism. However, favorable conditions for the formation of the M-phase are created by a homogeneous gas-phase mechanism. The nucleation of the M-phase could be favored by specific intermolecular interactions between carbon dioxide and dioxidine molecules. Thus, the growth of the M-phase content, with an increase in the CO_2_ flow from 1.4 × 10^16^ to 7.0 × 10^17^ molecules s^−1^ cm^−2^, is probably associated with an increase in the contribution of the homogeneous gas-phase mechanism to the processes of nucleation and crystal growth.

A further increase in the flow of the carrier gas (CO_2_) leads to the formation of a new polymorphic modification of dioxidine, the structure of which is difficult to decipher due to the significant broadening of the peaks on the x-ray diffractograms in view of the small size of the particles.

The dependence of the phase composition of cryochemically obtained dioxidine nanoforms on the carrier gas flow is probably due to the possible adsorption of CO_2_ molecules on the surface of the crystals of the M phase compared to the crystals of the T phase. This could be connected by the fact that, for the M phase, one of the N-oxide groups does not participate in the formation of hydrogen bonds; therefore, a system of free functional groups is created on the surface of the monoclinic phase, with which carbon dioxide molecules could be involved into specific intermolecular interactions. In turn, more efficient adsorption of CO_2_ molecules on the surface of the M phase crystals led to a decrease in its surface energy compared to the T phase. From the classical theory of nucleation, the following formula is known [42]:

$$J=\;C*exp\left(-\frac{\Delta G^{*}}{kT}\right);\;\;\;\Delta G^{*}=\;\frac{16}{3}\frac{\pi \;\sigma^{3}v_{1}^{2}}{{\left(kT\;lnS\right)}^{2}}$$

***J*** is the nucleation rate, ***C*** is the pre-exponential factor, which is slightly dependent on temperature and supersaturation, ***S*** is the supersaturation, ***σ*** is the surface energy, ***v*_1_** is the volume occupied by one molecule in condensed phase, and ***k*** is the Boltzmann constant. This formula implies an extremely sharp dependence of the rate of nucleation on the surface energy.

**Figure 6:**
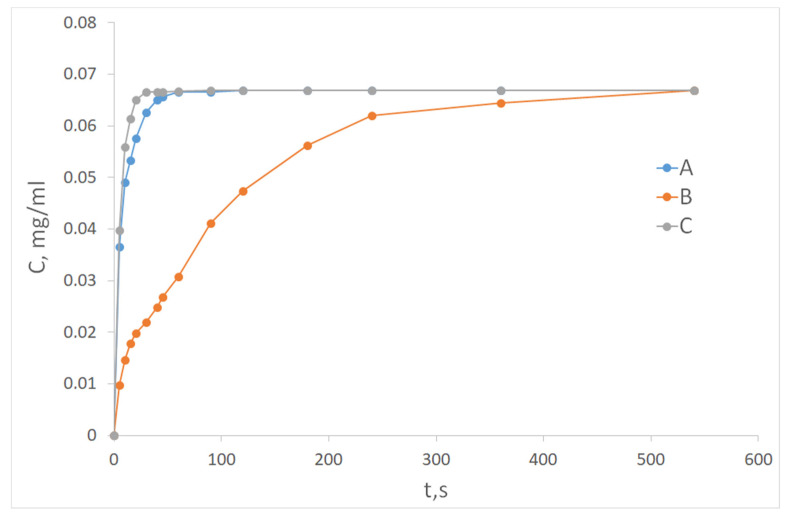
Kinetic curves of dissolution of dioxidine samples: A—dioxidine obtained at a minimal carrier gas flow (CO_2_), B—initial pharmacopoeia dioxidine, C—dioxidine obtained at a maximum carrier gas flow—6.97 × 10^17^ moleculs s^−1^ cm^−2^.

The rate of dissolution of dioxidine was determined using UV/Vis of spectrophotometry. The solubility curves of the initial and cryochemically modified dioxidine are shown in Figure 6. It takes about one hour for the initial dioxidine to completely dissolve. The rate of dissolution of cryochemical modified dioxidine is much higher. For example, dioxidine obtained in the absence of carrier gas flow completely dissolves in ~60 s, and dioxidine obtained at maximum carrier gas flow completely dissolves in ~30 s. In this case, the solubility curves of both cryochemically modified and initial dioxidine obey the kinetic law of the first order with good accuracy. The Noyes–Whitney equation is often used to describe kinetic solubility curves [43,44]:

$$\frac{dX}{dt}=\;\frac{D\;S}{h_{D}}*\;\left(C_{s}-C_{t}\right)$$

***dX***/***dt*** is the rate of dissolution, ***D*** is the diffusion coefficient, ***S*** is the effective surface area of the solute, ***h_D_*** is the characteristic diffusion size, ***C_s_*** is the concentration of the solute corresponding to saturation, and ***C_t_*** is the current concentration of the substance in the volume.

In the case when the concentration corresponding to the saturated solution is reached, the amount of substance remaining in the precipitate is much greater than the amount of the dissolved substance; it can be assumed that, during dissolution, the effective surface area of the particles of the dissolved substance remains constant. In this case, for a monodisperse sample, the Noyes–Whitney equation is a first-order kinetic equation with a velocity constant proportional to the parameter ***S***. This indirectly indicates a small dispersion of the particle size distribution.

Mathematical processing of the dissolution curves according to the kinetic law of the first order gives us the following values of the times when the current concentration of dioxidine is equal to half the concentration of the saturated solution. For the initial dioxidine, this time was 74 s, for cryochemically modified dioxidine obtained in the absence of CO_2_, it is 8.8 s, and for cryochemically modified dioxidine obtained at the maximal flow of CO_2_, this time is 5.3 s.

**Figure 7:**
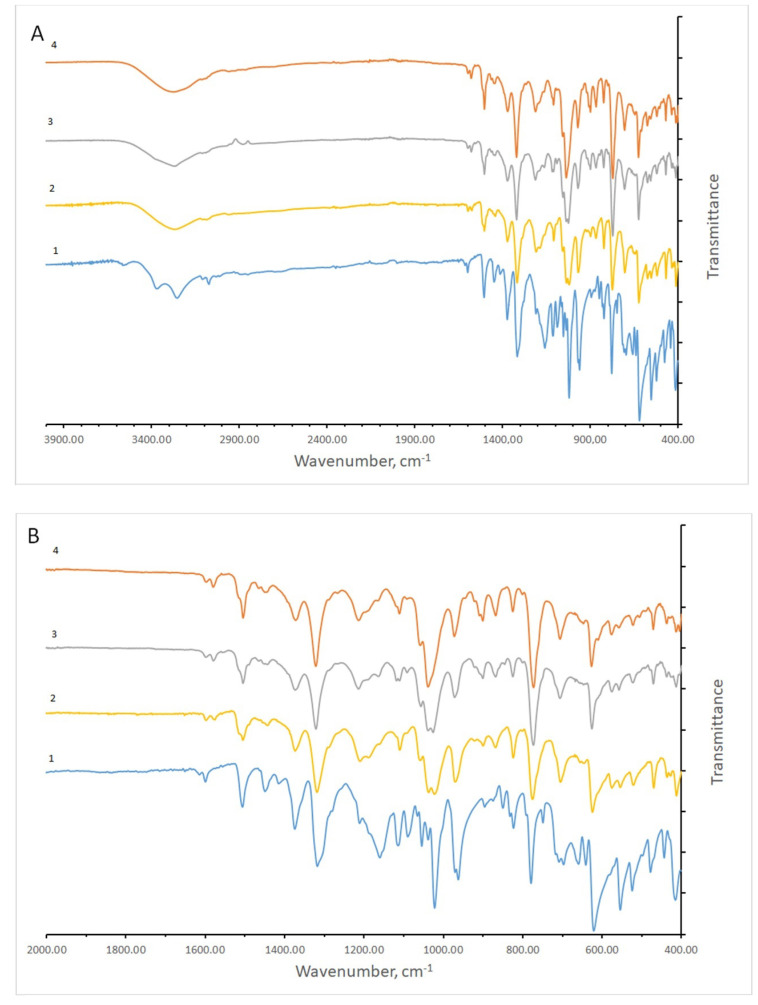
FTIR spectra in the ranges 400–4000 (**A**) and 400–2000 (**B**) cm^−1^ of initial pharmacopoeia dioxidine (1), dioxidine obtained at a minimal carrier gas flow (2), dioxidine obtained with a carrier gas (CO_2_) flow of 7.0 × 10^16^ (3), and dioxidine obtained at a maximum carrier gas flow of 6.97 × 10^17^ moleculs s^−1^ cm^−2^ (4).

As can be seen from Figure 7 that the spectra of cryomodified samples possess some features different from the spectrum of the initial dioxidine, which is due to the presence of water molecules in the crystal structure of the initial dioxidine monohydrate. Thus, for the initial dioxydine monohydrate (1:1, one molecule of water per one molecule of dioxidine), the bands of stretching vibrations of the O-H bonds of the hydroxyl group of crystal hydrate water molecule (band with maximum at 3350 cm^−1^) and the O-H bonds of the hydroxyl groups of dioxidine molecules (band with maximum at 3250 cm^−1^) differ, while for cryomodified samples, one band is observed (with a maximum at 3260 cm^−1^). The stretching vibration frequencies, as well as the deformation vibration frequencies of the OH bonds, are significantly shifted (by 500 cm^−1^) to the long-wave region due to the formation of a system of classical hydrogen bonds. The presence of water molecule in the structure of the initial dioxidine monohydrate appear as a wide band (with a maximum at 1160 cm^−1^), corresponding to the deformation vibrations of water OH-groups. This band (with a maximum at 1190 cm^−1^) is also presented for the sample of cryomodified dioxidine obtained at zero flow rate of carrier gas (CO_2_) due to the existence in the sample of small amounts of the 1/3 (one molecule of water per 3 dioxidine molecules) hydrate form (H-phase).

By comparing FTIR spectra of the initial and cryomodyfied dioxidine substance, the largest deviations were recorded in the region of the stretching C = C vibrations of the 1,2-disubstituted benzene ring (1615 cm^−1^, 1599 cm^−1^ for the initial dioxidine, 1599–1597 cm^−1^ and 1576–1577 cm^−1^ for the cryomodified dioxidine samples). Significant deviations are in the region of scissor vibrations −CH2-group (1450 cm^−1^ in the initial dioxidine, 1442–1444 cm^−1^ in the samples of cryomodified dioxidine). There are also noticeable deviations in the region of out-of-plane vibrations of the C-H group of the benzene ring (779 cm^−1^ in the initial dioxidine, 772–774 cm^−1^ in the samples of cryomodified dioxidine). In addition, the initial dioxidine form possess several bands that are absent in the samples of cryomodified dioxidine: 1159 cm^−1^, 1089 cm^−1^, 850 cm^−1^, and 749 cm^−1^ These changes can be connected with the conformational differences of dioxidine molecules and the changes in the system of hydrogen bonds stabilizing the T-, M-, and H-crystal forms belonging to cryo-modified samples. One of these differences appears as a band with a maximum at 1110 cm^−1^. It appeared in the case of cryomodified dioxidine samples obtained at a gas carrier (CO_2_) flow of 7 × 10^16^ molecules/(s cm^2^), (curve 2) due to the formation of the monoclinic crystal form (M-phase), which includes 2 types of N+-O-bonds (forming a non-classical H-bond and not forming one). For cryomodified dioxidine sample obtained at a zero flow of carrier gas (CO_2_) and consisting almost entirely of the triclinic crystal vibration frequencies form (T-phase), in which all N+-O-bonds are equivalent, the peak is not cleaved. The same cleavage was observed for the cryomodified dioxidine sample obtained at a carrier gas flow of 7 × 10^17^ molecules/(s cm^2^), which allows us to suggest that the unknown crystal phase (N-phase) is close to the monoclinic crystal structure.

**Figure 8:**
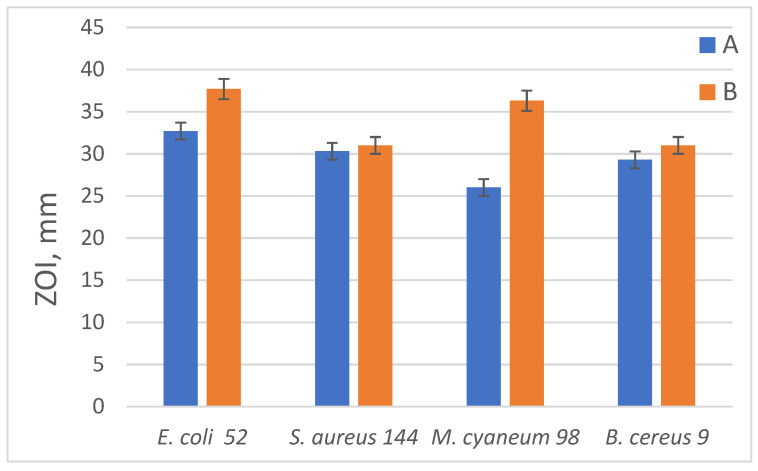
Zones of bacteria strains growth inhibition (ZOI) for compressed tablets of initial pharmacopoeia dioxidine (**A**) and dioxidine obtained at a maximum carrier gas flow of 6.97 × 10^17^ moleculs s^−1^ cm^−2^ (**B**).

The data on the antibacterial activity of cryomodified dioxidine forms against *E. coli 52*, *S. aureus 144*, *M. cyaneum 98*, and *B. cereus 9* were obtained by disc-diffusion procedure (see Section 2.3.7). The diameters of the zones of inhibition of bacterial strains growth (ZOI) were measured and are presented in Figure 8 and Table S1. It was found that, around the non-modified dioxidine tablets, ZOI values were 5–10 mm less than that for cryomodified dioxidine in the case of *E. coli* and *M. cyaneum* cells, and 1.4–1.7 less than that for cryomodified dioxidine in the case of *S. aureus* and *B. cereus.* The difference between the antibacterial effect of cryomodified and pharmacopoeias drugs (ZOI values) on *S. aureus 144 and B.cereus 9* bacterial strains is rather lower than for *E. coli 52 and M. cyaneum 98*, which could be connected with the specific mechanisms of different bacteria cells damage by this antibiotic substance. Therefore, these experiments showed that the bactericide activity of cryomodified dioxidine obtained at a maximum carrier gas flow of 6.97 × 10^17^ moleculs s^−1^ cm^−2^ was higher in comparison with the initial pharmacopoeia dioxidine. The sequence of the antibacterial activity data for the different dioxidine forms correlates well with the changes in particle size and dioxidine crystal phase composition of cryomodified patterns controlled by varied carrier gas (CO_2_) flow.

## 4. Conclusions and Outlook

The combined cryochemical method for drug nanoform preparation has been proposed. It includes drug substance conversion in the gas (vapor) phase due to the sublimation process and its combination with the flow of an inert carrier gas, carbon dioxide (CO_2_), followed by low-temperature condensation of the gas/vapor mixture on a cooled-by-liquid nitrogen support surfaces. Using this method, nanoforms of the antibacterial drug 2,3-quinoxalinedimethanol-1,4-dioxide (dioxidine) has been obtained that is identical in chemical composition to the original pharmacopoeia dioxidine (according to UV/Vis spectrophotometry), but different in the particle size and solid phase crystal structure. Obtained FTIR-data show the changes due to the cryomodification of dioxidine, which can be connected to the conformational differences of dioxidine molecules and changes in the system of hydrogen bonds stabilizing T-, M-, and H-crystal forms belonging to cryomodified samples.

It was found that, according to powder XRD, the crystal phase composition of the obtained cryomodified dioxidine samples is dependent on the value of the carrier gas (CO_2_) flow rate. It was shown that the obtained cryomodified samples consisted of two anhydrous triclinic (T-phase), one monoclinic (M-phase), and one 1/3 crystal hydrate (H-phase) (one water molecule per 3 dioxidine molecules) phases. At high values of the carrier gas flow, a new unidentified crystal phase of cryomodified dioxidine also appeared that was morphologically close to the structure of the monoclinic crystal phase (M-phase). The increase in the flow rate of the gas carrier (CO_2_) led to a decrease in the average particle size of the drug particles and to changes in the crystal phase composition of cryomodified samples. It was found that dissolution rates of cryomodified dioxidine in pure water and water solutions exceeded those of the original ones 8–14 times. Cryomodified dioxidine samples possessed statistically improved antibacterial activity in comparison to the initial pharmacopeia product against *E. coli 52* and *M. cyaneum 98*, while less significant, but statistically reliable, results were obtained for the zones of inhibition (ZOI) of bacterial cells growth for *S. aureus 144* and *B. cereus 9*.

Thus, the results obtained in this work show the possibility of antibacterial drug dioxidine nanoforms obtained by the combined cryochemical modification method and by controlling the particle size and crystal phase composition by varying the gas carrier (CO_2_) flow rate.

## Figures and Tables

**Table 1:** Size and structural characteristics of cryochemically modified dioxidine obtained using combined gas-phase condensation method.

Flow of CO_2_, Mole/h	Condensation Rate of Carrier Gas CO_2_, molecules s^−1^ cm^−2^ × 10^−16^	The Ratio of Condensation Rates CO_2_/Dioxidine	Phase Composition	Specific Surface Area, m^2^/g	Particle * Size, nm
0	0.0	0	T:H = 7:1	9.6	414 ± 80
0.2	1.4	1.85	T:M = 2:1	33	120 ± 24
0.45	3.1	4.16	T:M = 1:1	34	118 ± 23
1.00	7.0	9.24	T:M = 2:3	37	108 ± 22
4.5	31.4	41.63	T:N = 3:2	71	56 ± 11
10.0	69.7	92.50	T:N = 3:2	78	51 ± 10

* Measured by chromatographic determination of the specific surface area of the samples using low temperature adsorption of inert gas argon, followed by its desorption during thermos-programmed heating (see Section 2.3.5).

## Supplementary Materials

The following are available online at https://www.mdpi.com/article/10.3390/nano11061588/s1: Figure S1. The scheme of the original cryostat for the cryochemical synthesis using a combination of sublimation of dioxidine drug and joint low-temperature condensation of its molecular beam with the flow of inert gas-carrier; Figure S2, Fragment of the crystal lattice of dioxidine monohydrate (SOKGAA); Figure S3, Simulated X-ray diffraction pattern of dioxidine monohydrate (SOKGAA); Figure S4, Fragment of the crystal lattice of monoclinic dioxidine polymorph (M); Figure S5. Simulated X-ray diffraction pattern of monoclinic dioxidine polymorph (M). Figure S6, Fragment of the crystal lattice of triclinic dioxidine polymorph (T); Figure S7, Simulated X-ray diffraction pattern of triclinic dioxidine polymorph (T); Figure S8, Fragment of the crystal lattice of 1/3 dioxidine hydrate (H); Figure S9, Simulated X-ray diffraction pattern of 1/3 dioxidine hydrate (H); Table S1, Zones of bacteria strains growth inhibition (ZOI) for compressed tablets of initial pharmacopoeia dioxidine and dioxidine obtained at a maximum carrier gas flow of 6.97 × 10^17^ moleculs s^−1^ cm^−2^ (results were obtained by a nine-fold repetition measurements of the zones inhibition (ZOI) of bacterial growth for each series of samples).
