# Supplementary material for: Cryochemical Production of Drug Nanoforms: Particle Size and Crystal Phase Control of the Antibacterial Medication 2,3-Quinoxalinedimethanol-1,4-dioxide (Dioxidine)
Source: Nanomaterials (Basel). 2021 Jun 17;11(6):1588. doi: 10.3390/nano11061588 (PMC8235636; doi:10.3390/nano11061588)
Supplement: Supplementary file 1 [file nanomaterials-11-01588-s001.zip › nanomaterials-1258398-supplementary.pdf]

nanomaterials

MDPI

Supplement materials for the paper to Nanomaterials

Cryochemical Production of Drug Nanoforms: Particle Size and Crystal Phase Control of the Antibacterial Medication 2,3-Quinoxalinedimethanol-1,4-dioxide (Dioxidine)

Tatyana I. Shabatina [1,2,*], Yurii N. Morosov [1,2], Andrey V. Soloviev [1], Andrey V. Shabatin [3], Olga I. Vernaya [1] and Michail Y. Melnikov [1]

1 Department of Chemistry, M.V. Lomonosov Moscow State University, Leninskie Gori build 1/3, 119991 Moscow, Russian Federation; yunmor@mail.ru (Y.N.M.); andr_solovyov@mail.ru (A.V.S.); olga_vernaya@mail.ru (O.I.V.); melnikov46@mail.ru (M.Y.M.)

2 Department of Chemistry, Faculty of Fundamental Sciences, Bauman Moscow State Technical University, 2nd Bauman Str. 5, 105905 Moscow, Russian Federation

3 A.N. Frumkin Institute of Physical Chemistry and Electrochemistry RAS, Lenin Prospect, 31 build. 4; 119071 Moscow, Russian Federation, 5dr.on5@mail.ru (A.V.Sh)

* Correspondence: tatyanashabatina@yandex.ru or tsh@kinet.chem.msu.ru; Tel./Fax: +07-495-939-5442

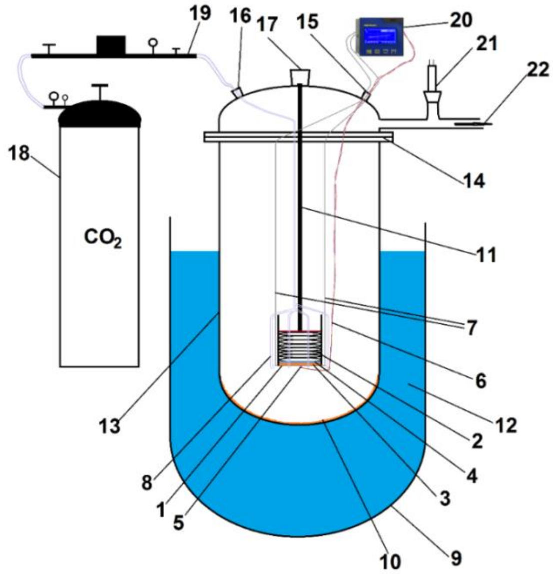

Figure S1. The scheme of the original cryostat for the cryochemical synthesis using combination of sublimation of dioxidine drug and joint low-temperature condensation of its molecular beam with the flow of inert gas-carrier: 1 - a metal grid heated by electric current flowing through it; 2 - spring that presses the layer of the original drugs to the metal grid; 3 - a layer of the original drug substances pressed against a metal grid; 4 - a rod that serves for evenly pressing the layer of the initial drugs to the metal grid; 5 - junction of a thermocouple used for measuring the temperature of a metal grid; 6 – copper - constantan thermocouple designed for measuring the temperature of a metal grid; 7 - network wires connected to a metal grid; 8 - comb designed for uniform distribution of the flow of carrier gas-CO_2; 9 - Dewar vessel with liquid nitrogen; 10 - layer of a mixture of a cryomodified drugs with solid carbon dioxide; 11 - pin that regulates the position of the sublimator in the height of the submerged reactor; 12 - liquid nitrogen; 13 - vacuum-operated submersible reactor; 14 – movable vacuum connection; 15 - vacuum input for thermocouple and network wires; 16 - vacuum inlet for CO_2 carrier gas flow; 17 - vacuum input for dioxide; 19 - gas main (gas flow regulator); 20 - temperature controller; 21 - vacuum sensor; 22 - access to the vacuum system the pin that regulates the position of the sublimator in the height of the submerged reactor; 18 - metal cylinder with carbon.

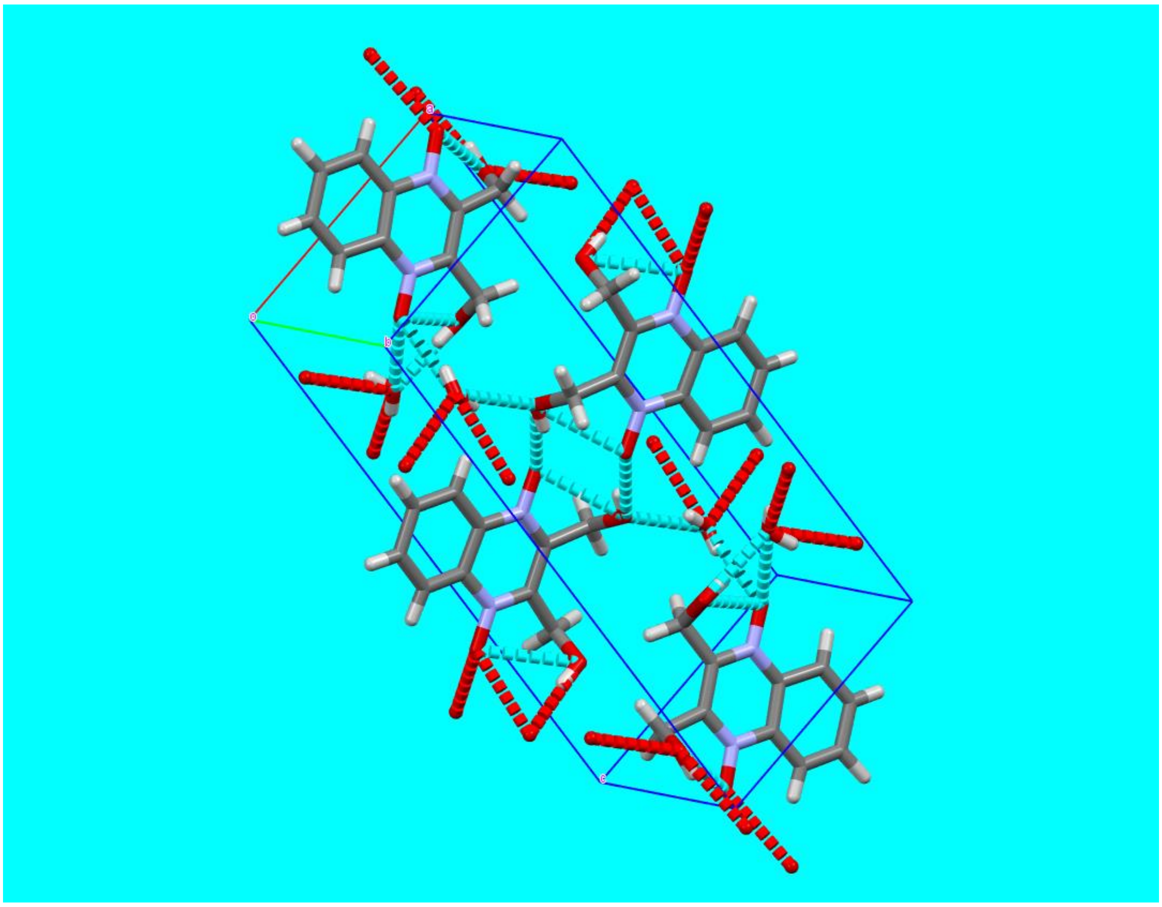

Figure S2. Fragment of the crystal lattice of dioxidine monohydrate (SOKGAA).

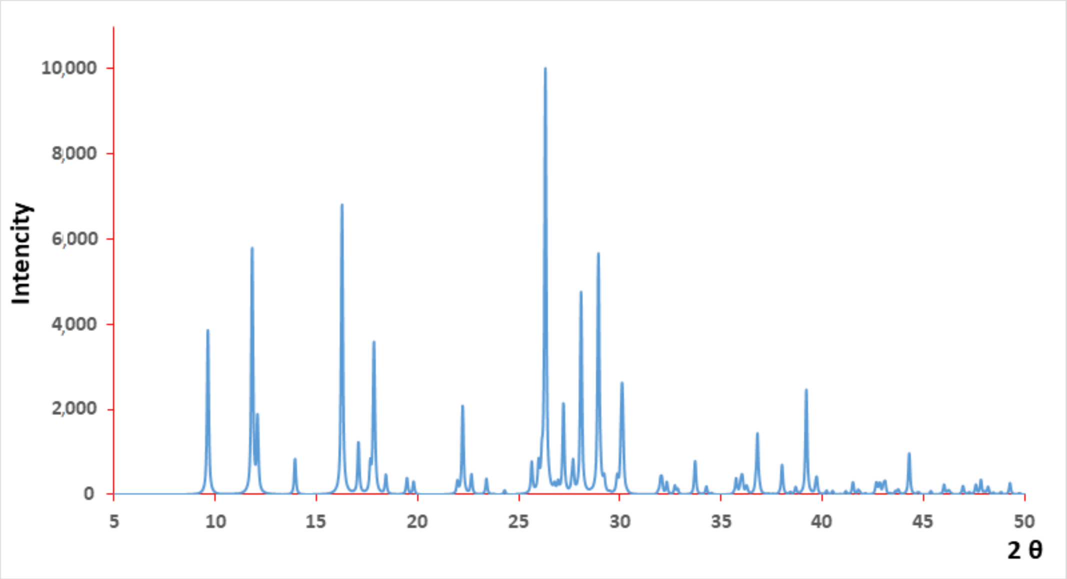

Figure S3. Simulated X-ray diffraction pattern of dioxidine monohydrate (SOKGAA) [40].

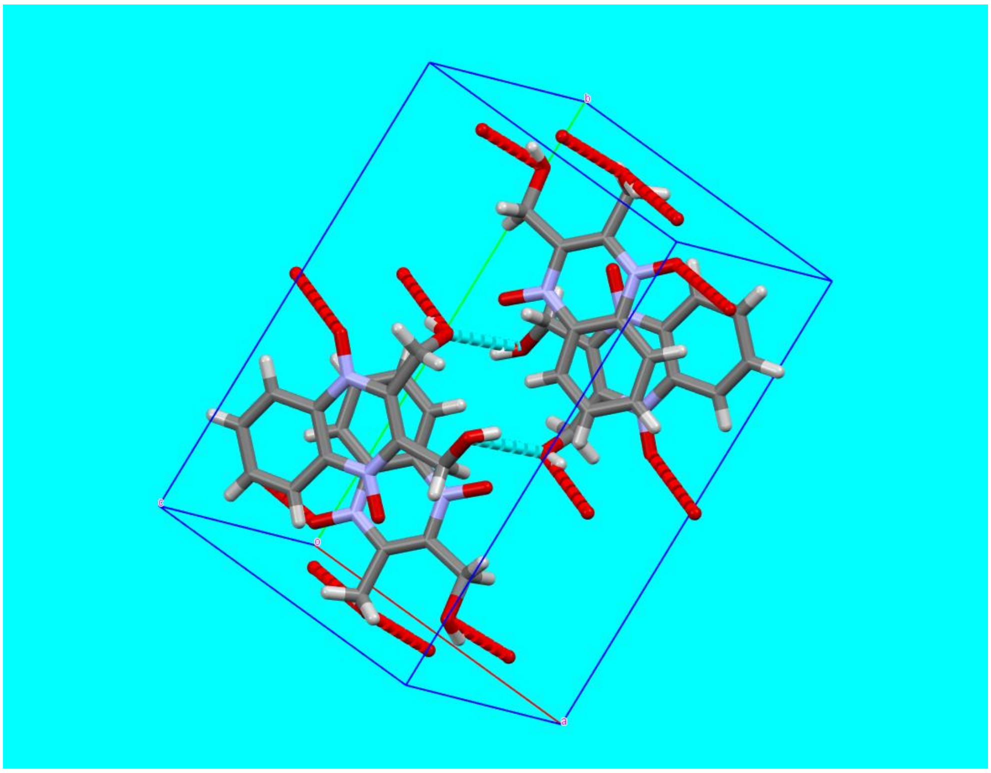

Figure S4. Fragment of the crystal lattice of monoclinic dioxidine polymorph (M).

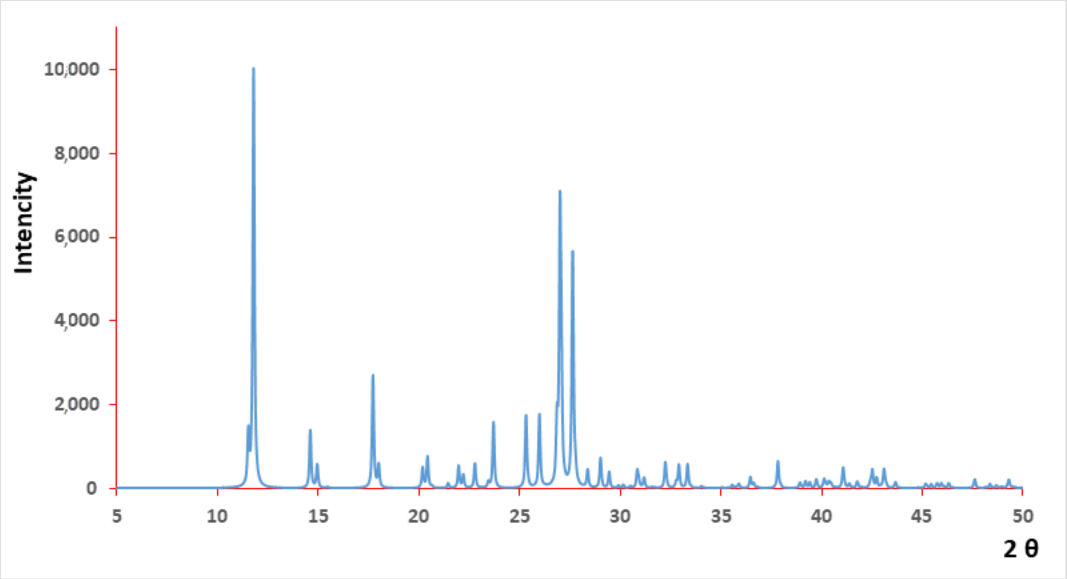

Figure S5. Simulated X-ray diffraction pattern of monoclinic dioxidine polymorph (M) [39].

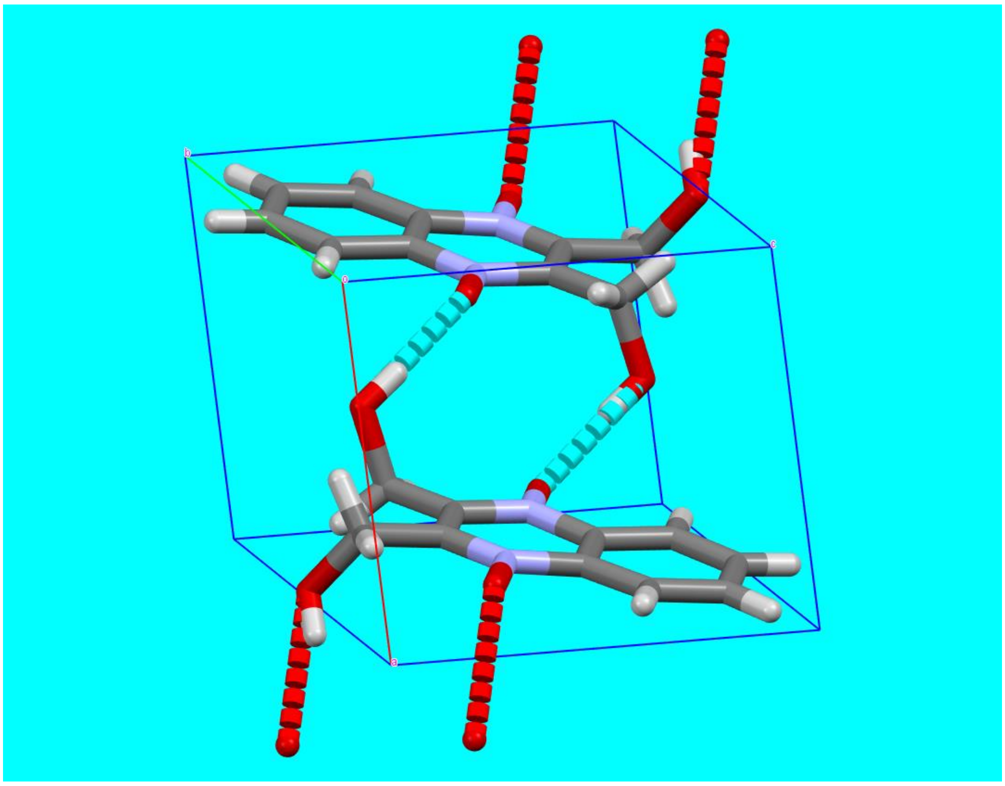

Figure S6. Fragment of the crystal lattice of triclinic dioxidine polymorph (T).

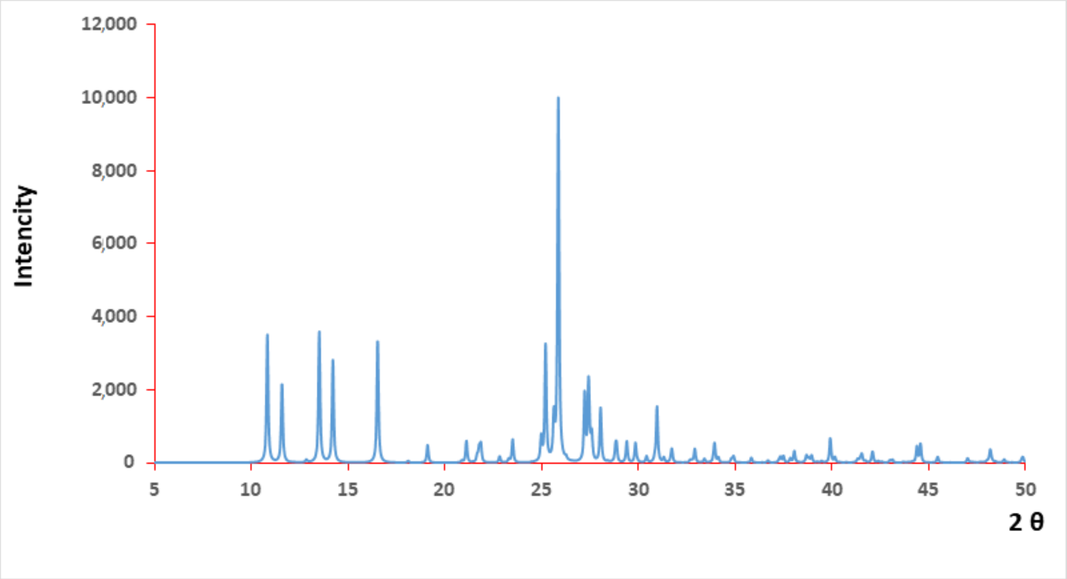

Figure S7. Simulated X-ray diffraction pattern of triclinic dioxidine polymorph (T) [39].

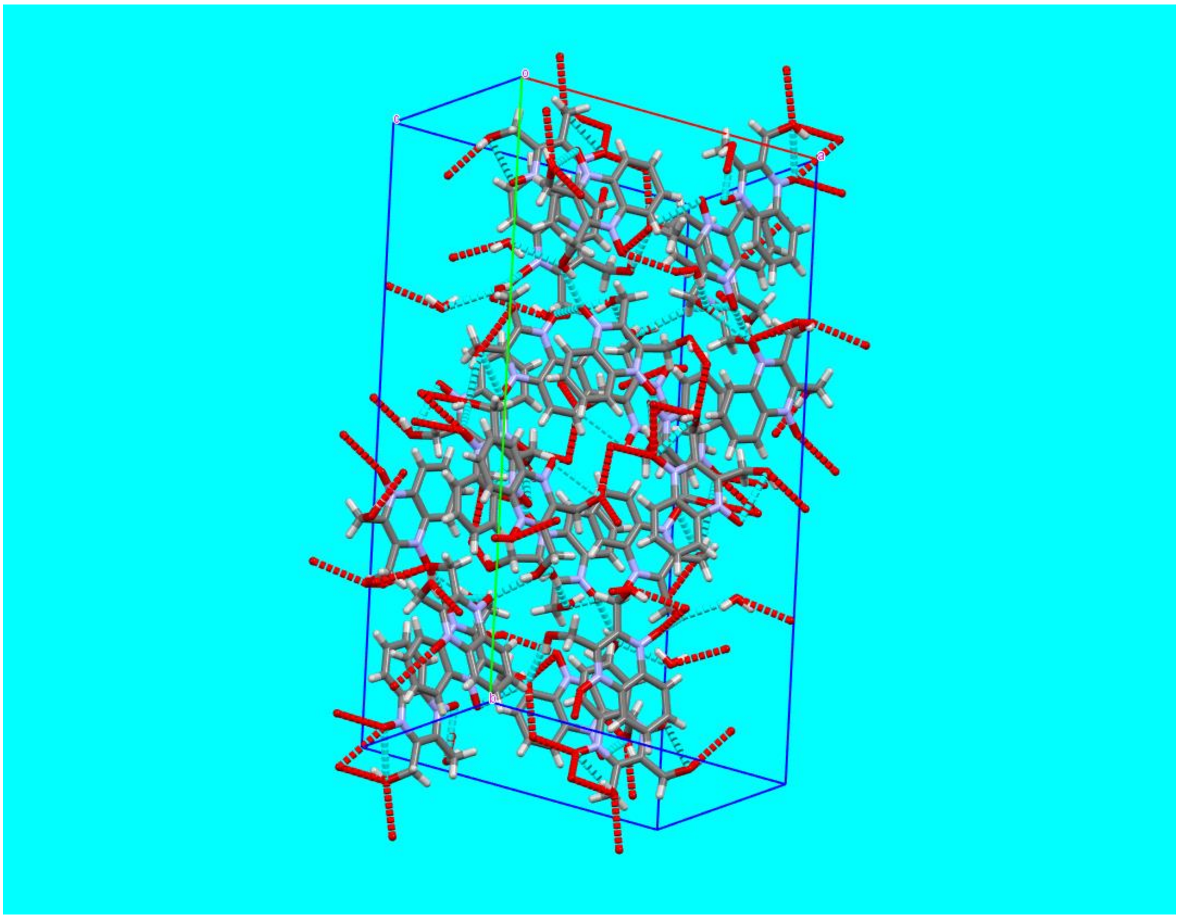

Figure S8. Fragment of the crystal lattice of 1/3 dioxidine hydrate (H).

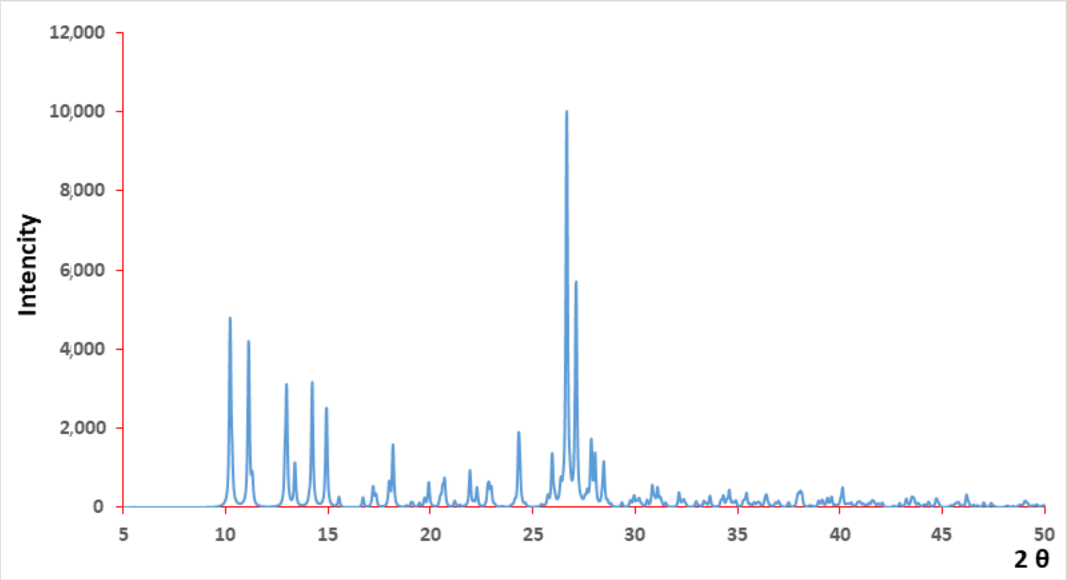

Figure S9. Simulated X-ray diffraction pattern of 1/3 dioxidine hydrate (H) [39].

Table S1. Zones of bacteria strains growth inhibition (ZOI) for compressed tablets of initial pharmacopoeia dioxidine and dioxidine obtained at a maximum carrier gas flow–6,97*1017moleculs*s^{-1}*cm^{-2} , results were obtained by a nine-fold repetition measurements of zones of inhibition (ZOI) of bacterial strains growth for each series of samples.

Bacteria Strains	Drug Substance	ZOI (mm)	Mean (mm)	Standard Deviation (mm)	p-Value
E coli 52	Pharmacopoeia dioxidine	32	32.7	1.0	5.7×10^{-8}
		33			
		31			
		34			
		33			
		32			
		34			
		33			
		32			
	Cryomodified Dioxidine obtained at a maximal carrier gas CO_2 flow (7*10^{17} molecules*s^{-1}*cm^{-2})	39	37.7	1.2	
		35			
		37			
		39			
		38			
		38			
		37			
		38			
		38			
S. aureus 144	Pharmacopoeia dioxidine	29	30.3	0.9	8.1×10^{-3}
		30			
		30			
		32			
		30			
		30			
		30			
		31			
		31			
	Cryomodified Dioxidine obtained at a maximal carrier gas CO_2 flow (7*10^{17} molecules*s^{-1}*cm^{-2})	32	31.7	1.0	
		31			
		31			
		31			
		32			
		30			
		33			
		32			
		33			

M. cyaneum 98	Pharmacopoeia dioxidine	26	26.0	0.9	5.8×10^−13^
		25			
		25			
		27			
		27			
		27			
		26			
		25			
		26			
	Cryomodified Dioxidine obtained at a maximal carrier gas CO_2 flow ($7*10^{17}$ molecules$*s^{-1}*cm^{-2}$)	38	36.3	1.2	
		37			
		37			
		35			
		36			
		38			
		35			
		35			
		36			
B. cereus 9	Pharmacopoeia dioxidine	30	29.3	1.0	$2.7×10^{-3}$
		31			
		30			
		29			
		29			
		28			
		29			
		28			
		30			
	Cryomodified Dioxidine obtained at a maximal carrier gas CO_2 flow ($7*10^{17}$ molecules$*s^{-1}*cm^{-2}$)	29	31.0	1.0	
		31			
		31			
		32			
		32			
		31			
		30			
		32			
		31			
